# A Gigantic Shark from the Lower Cretaceous Duck Creek Formation of Texas

**DOI:** 10.1371/journal.pone.0127162

**Published:** 2015-06-03

**Authors:** Joseph A. Frederickson, Scott N. Schaefer, Janessa A. Doucette-Frederickson

**Affiliations:** 1 Department of Biology, University of Oklahoma, Norman, Oklahoma, United States of America; 2 Department of Geosciences, University of Wisconsin-Milwaukee, Milwaukee, Wisconsin, United States of America; 3 Department of Anthropology, University of Oklahoma, Norman, Oklahoma, United States of America; Team 'Evo-Devo of Vertebrate Dentition', FRANCE

## Abstract

Three large lamniform shark vertebrae are described from the Lower Cretaceous of Texas. We interpret these fossils as belonging to a single individual with a calculated total body length of 6.3 m. This large individual compares favorably to another shark specimen from the roughly contemporaneous Kiowa Shale of Kansas. Neither specimen was recovered with associated teeth, making confident identification of the species impossible. However, both formations share a similar shark fauna, with *Leptostyrax macrorhiza* being the largest of the common lamniform sharks. Regardless of its actual identification, this new specimen provides further evidence that large-bodied lamniform sharks had evolved prior to the Late Cretaceous.

## Introduction

Shark (Chondrichthyes; Elasmobranchii) vertebrae are less common than teeth in the fossil record, and unlike teeth, are relatively undiagnostic at the species level when found in isolation. Nevertheless, isolated vertebrae are scientifically useful as they produce more accurate total body length estimates than do individual teeth [1,2]. Thus, shark vertebrae yield important data on the biology and ecology of fossil sharks, even without clear species association. Here we describe three isolated vertebrae (Sam Noble Oklahoma Museum of Natural History [OMNH] 68860) from a very large lamniform shark found within Albian-age rocks of north-central Texas. Taxonomic identification is difficult, as no teeth were recovered in association with the vertebrae. However, these specimens are important because they represent some of the largest published lamniform shark vertebrae from the Early Cretaceous of North America.

### Geologic setting

**Fig 1:**
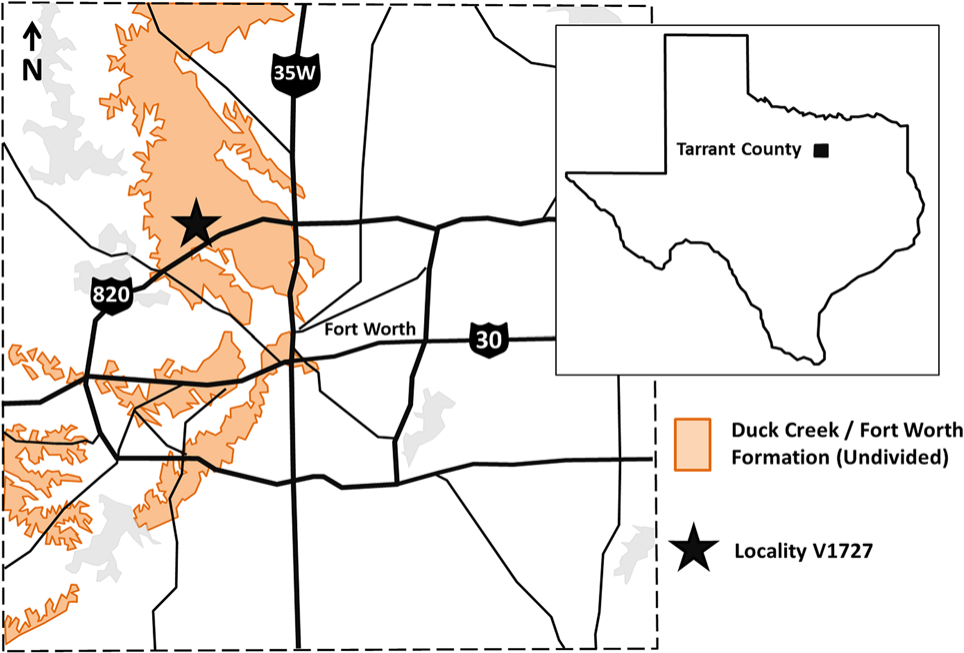
A map of Albian-age rocks in Tarrant County, Texas, showing the approximate location of OMNH V1727.

The specimens described herein, OMNH 68860, were collected in the Duck Creek Formation of Tarrant County, Texas (Fig 1). The Duck Creek is the second lowest formation of the Lower Cretaceous Washita Group (Albian)[3]. Underlying the Duck Creek is the basal Kiamichi Formation, which shares a contact with the Duck Creek defined lithologically by a transition from pebble-conglomerates and breccias to limestone, and faunally by a sharp decrease in *Gryphaea* and *Schloenbachia* [4, 5]. The Duck Creek is conformably overlain by the Fort Worth Formation; the intervening contact is lithologically inconspicuous but is instead marked biostratigraphically by the appearance of *Holaster simplex*, *Hemiaster elegans*, and *Exogyra americana* [5].

Within Tarrant County, the Duck Creek Formation is approximately 13 m thick and is comprised of limestone, marl, and chalky marl deposits exposed in the western half of the county (Fig 1) [4]. The Duck Creek Formation is subdivided into two primary units based on lithology, and four primary zones based on fauna. Lithologically, the first 7 m above the base of the Duck Creek are dominated by limestone. Above 7 m, limestone beds become increasingly indurated, decrease in thickness, and are further interlaminated by marl or marly limestone [4, 6]. Biostratigraphic zones consist of three ammonoid faunas including the basal *Desmoceras* zone, followed by the *Schloenbachia* zone and a *Scaphites* zone; the uppermost faunal zone is marked by the appearance of the brachiopod genus *Kingena* [4].

**Fig 2:**
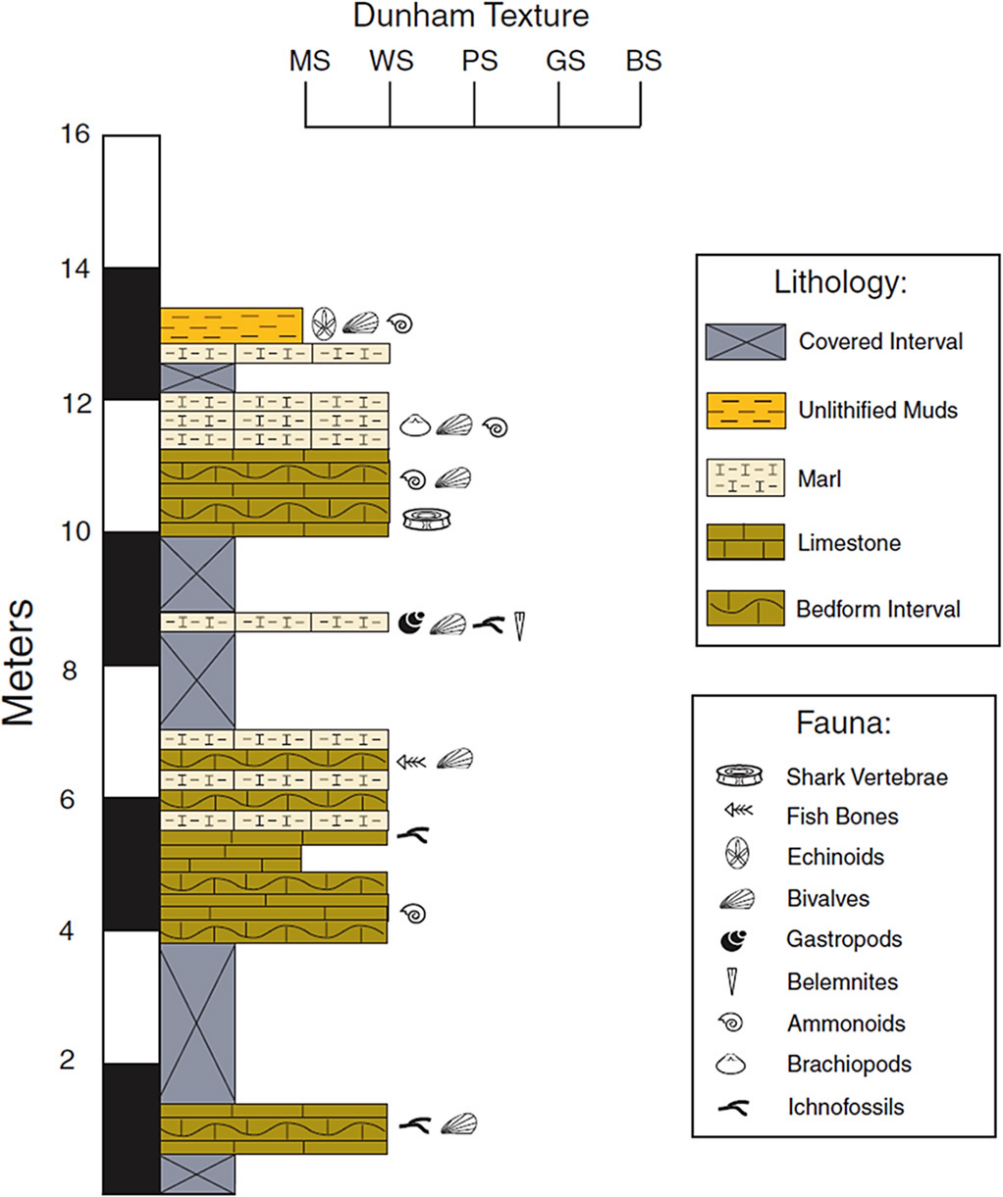
A stratigraphic column at V1727.

The base of a measured section at V1727 begins within the Duck Creek Formation and correlates both lithologically and faunally with the ammonoid-rich limestone beds as described by Winton and Adkins [4] (Fig 2). Limestone dominates the measured section from the base to 6 m, where the first thinly-bedded marl deposits occur. Despite significant portions of the section being covered by talus, the lithological transition to marl-dominated strata above 6 m is clearly defined and further supported by the appearance of *Kingena* among the talus. The uppermost strata of the measured section consist of weathered, *Kingena*-bearing marl with various echinoid fragments belonging to *Holaster simplex*. These taxa imply the transition to the overlying Fort Worth Formation; however, no *in situ* bedforms were observed; thus, at present, the contact can be inferred but not directly observed.

**Fig 3:**
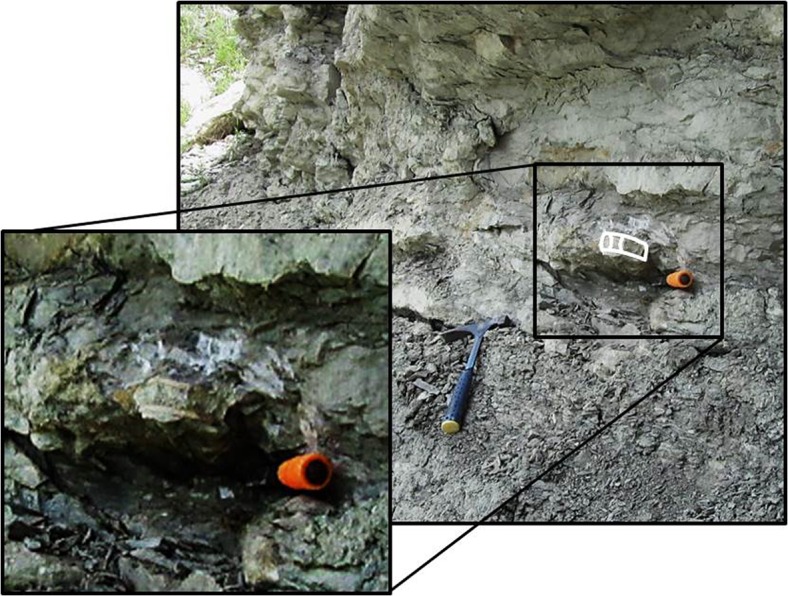
Additional shark vertebrae found *in situ* in the same locality as OMNH 68860. The surrounding lithology correlates with the indurated limestone bedforms 10.5 m above the base of the measured section (photo courtesy of L. Hall, 2013).

Initially, the three vertebrae discussed herein were collected from a displaced block of limestone resting among marl debris situated above the lower limestone strata of the Duck Creek Formation. Further specimens bearing similar dimensions, taphonomic characteristics, and proximity to OMNH 68860 were recovered *in situ* by a private collector within the indurated limestone beds lying just below the *Kingena*-bearing marls approximately 11.5 m above the base of section (Fig 3; L. Hall, personal communication). The lithology of the strata containing the shark vertebrae found *in situ* matches the remaining matrix from OMNH 68860. Furthermore, taphonomic similarities shared among all vertebrae indicated that the specimens recovered by the private collector must indeed represent one individual (discussed below). The stratigraphic origin of the shark vertebrae can therefore be confidently placed below the lower *Kingena*-bearing marl and within the indurated limestone of the upper Duck Creek Formation, approximately 10.5 m above the base of the section.

## Materials and Methods

### Size estimation

In order to estimate the total body lengths from individual vertebra, we made comparisons with Cretaceous sharks that possess a more robust postcranial fossil record. As it is impossible to determine vertebral position in a shark based on isolated vertebrae alone [1], we conservatively regard the vertebra with the maximum centrum diameter as the largest vertebra in the entire individual. This ensures that the estimates achieved represent the smallest hypothetical length possible for this specimen. Additionally, we assume that the relationship between vertebral size and total body length is consistent between well-represented species and OMNH 68860. This assumption is reasonable because most pelagic sharks have a consistent body form [2].

Shimada [2] created a formula to estimate total body length from individual vertebrae in *Cretoxyrhina mantelli*. The relationship between centrum diameter (*CD* in mm) and total length (*TL*) can be estimated with the following formula:

TL(cm)=0.281+5.746(CD)

Similarly, Gottfried et al. [7] used a different formula to calculate the total body length of individual vertebra in *Carcharocles megalodon* using *Carcharodon carcharias* as a proxy.

TL(m)=0.22+0.058(CD)

We calculated total lengths for OMNH 68860 using both formulas.

### Permits

No permits were required for the described study, which complied with all relevant regulations.

## Results and Discussion

### Systematic Paleontology

- Class CHONDRICHTHYES Huxley 1880 [8]
- Subclass ELASMOBRANCHII Bonaparte 1838 [9]
- Order LAMNIFORMES Berg 1958 [10]
- Family, Genus and Species Indet.

#### Specimens

OMNH 68860; three vertebral centra, discovered by members of the Paleontology Club of the University of Wisconsin-Milwaukee, and prepared and curated at the Sam Noble Oklahoma Museum of Natural History in Norman, Oklahoma. Specimens were prepared by K. Davies at SNOMNH using a 10% buffered acetic acid bath for two of the three specimens. Additional vertebrae have also been recovered from the same site, but these specimens were not collected by the authors and are currently in a private collection.

#### Geologic Occurrence

Indurated limestone interval of the Duck Creek Formation (Lower Cretaceous: Washita Group) of north-central Texas.

#### Locality

The vertebrae were recovered at OMNH locality V1727, northwest of Fort Worth, Tarrant County, Texas (Fig 1). Locality data are on file at OMNH and are available upon request from qualified investigators.

### Description

**Fig 4:**
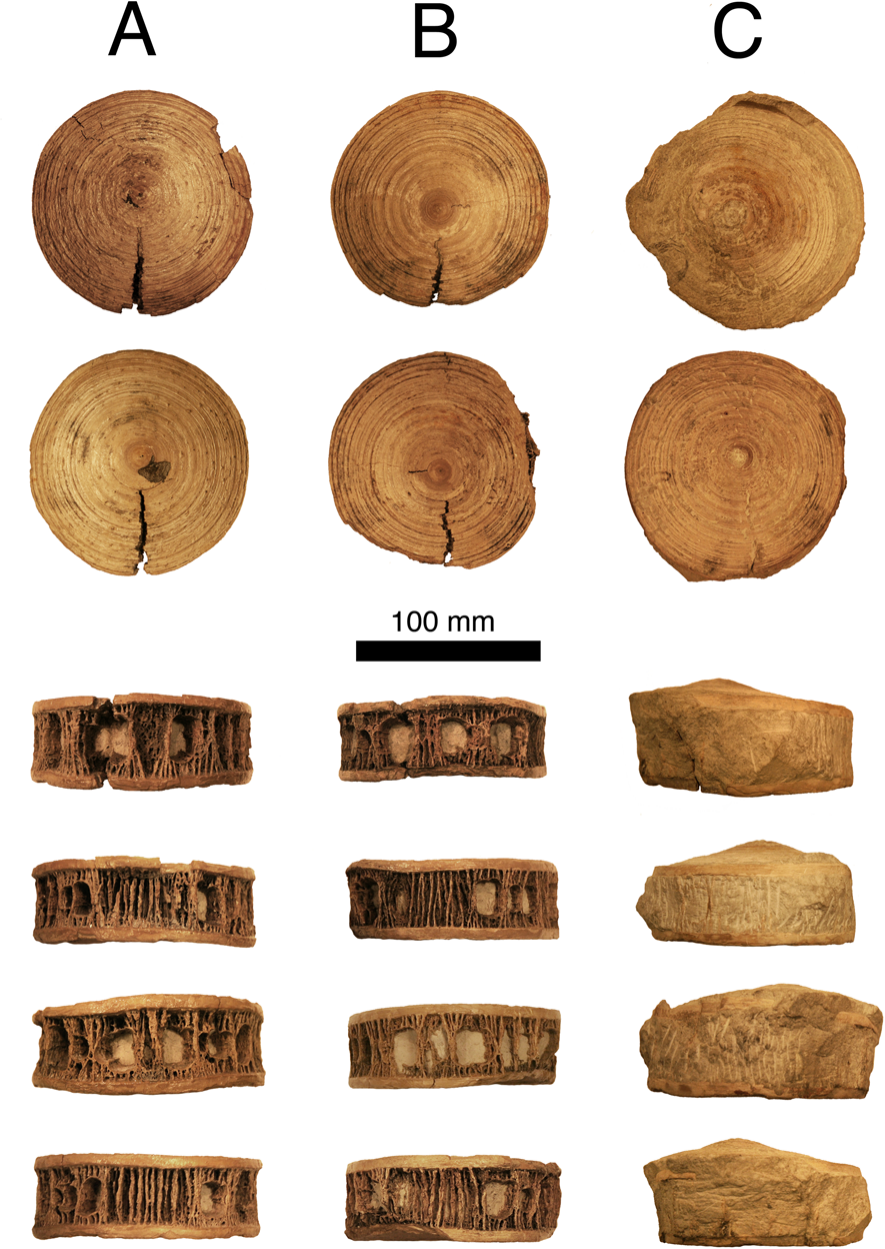
OMNH 68860 in (descending order) rostral, caudal, ventral, right lateral, dorsal, and left lateral views.

When discovered, all three vertebrae were disarticulated and were separated by a thin layer of limestone. The centra were recovered in a single vertical stack, with one vertebra situated perpendicular to the other two. All three are approximately the same size and proportions, implying that they represent vertebrae from the same area of the body. The largest, most-rostral vertebra measures approximately 110 mm in diameter, with a width of 34 mm (Fig 4). All three are rostrocaudally biconcave (amphicoelous) and roughly spherical in outline, with little to no deformation as a result of crushing. Both of the articular surfaces possess well-marked concentric lamellae on each vertebra. As in other lamniform sharks, all three vertebrae have multiple thin, radial lamellae circumventing at high densities around the outer surface of each centra. These lamellae measure up to 1.3 mm in diameter and run rostrocaudally with occasional bifurcations (Fig 4 right and left lateral view). Both articular surfaces possess a well-developed corpus calcareum, with a thickness measuring approximately 6 mm each.

Each vertebra possesses readily distinguishable cartilage foramina on both the dorsal and ventral surfaces. The dorsal surface was identified using techniques described by Shimada et al. [11], where the midline foramina with the smallest inter-pit distance in each centrum are designated the basidorsal cartilage foramina. Surprisingly, each of the two prepared vertebrae has multiple dorsal and ventral foramina (five ventrals, seven dorsals each). The two foramina on the midline are generally the largest; however, most do not extend to contact the corpus calcareum. All foramina are generally square with rounded corners and have a relatively smooth septum traveling into the vertebra. The additional foramina on the dorsal and ventral surface range in size, from 5.2–16.0 mm wide.

### Taphonomy

There were no other shark fossils associated with OMNH 68860. However, all three possessed traces of encrusting ostreids on the articular surfaces, indicating that these vertebrae were exposed at the surface for some period of time. Further, pyrite has developed near the center of both articular surfaces on the largest vertebra. The smallest of the three vertebrae retains a cast of the next vertebra’s corresponding articular surface, giving this specimen a concavo-convex appearance in lateral view. The three centra also possess a single radial fracture (37 mm long in each vertebra). The common location of this fracture indicates that breakage occurred prior to the rearrangement of the vertebrae. Although it cannot be completely determined whether OMNH 68860 represents reworked material, abrasion is minimal, indicating that transportation was not extensive.

### Size

Using the formulas of Shimada [2] and Gottfried et al. [7], and the maximum vertebral diameter of 110 mm, the minimum total length of the individual represented by OMNH 68860 is calculated to be 6.3 and 6.6 m respectively. According to these calculations, OMNH 68860 would rival the largest *Cretoxyrhina mantelli* specimens (6–7 m) [2] in total length and approximately equal the length of the largest documented extant great white shark (*Carcharodon carcharias*) (6.4 m) [12].

Although relatively large, OMNH 68860 are not the largest shark vertebrae known from the Early Cretaceous of North America. Shimada [13] described an isolated and incomplete large lamniform shark centrum, Kansas University Vertebrate Paleontology (KUVP) 16343, from the Kiowa Shale of Kansas. This incomplete specimen has a conservative estimated diameter of between 144–170 mm and a calculated complete body length of 8.3–9.8 m. Although smaller, OMNH 68860 compares favorably in appearance to the preserved portions of KUVP 16343 [13]. Shimada [13] tentatively assigned KUVP 16343 to the paraphyletic family Cretoxyrhinidae based on the large size of the specimen and the known presence of other ‘cretoxyrhinid’ sharks from the Kiowa Shale, *Cretalamna appendiculata* and *Leptostyrax macrorhiza* (recently reclassified as a member of family Eoptolamnidae [14]); both of which are also found in the Duck Creek Formation of Texas and Oklahoma [15]. Given their large size and the overlapping shark fauna between the two formations in which they were found, we hypothesize that the Kiowa and Duck Creek specimens are from the same species.

Both OMNH 68860 and KUVP 16343 represent relatively large Mesozoic lamniform sharks; however neither specimen would be considered extraordinary compared to some Cenozoic species. For example, an associated specimen of *Carcharocles angustidens* from the Late Oligocene has an estimated total body length of 6.6 – 9.3 m [16]; roughly matching that OMNH 68860 and KUVP 16343. Neither species, however, comes close to the maximum length estimates for the largest lamniform shark, *Carcharocles megalodon*. Total length estimates for this species vary depending on the method, but multiple techniques yield a gigantic size ranging from 9.2–16 m [3].

### Taxonomy

Morphological similarities between the Kiowa and the Duck Creek sharks are further supported by the age of their respective assemblages. Resemblances between the Early Cretaceous marine faunas of Texas and Kansas have long been recognized [17], and more recent studies have indicated that the Kiowa fauna correlates with that of the uppermost Fredericksburg Group and the lower Washita Group of Texas [18]. The index fossil *Inoceramus comancheanus* is found in the lower Duck Creek Formation and the middle Kiowa Shale, demonstrating that at least some of the Kiowa Shale is equivalent in age to the Duck Creek Formation. Based on their stratigraphy, the Kiowa Shale and Duck Creek have both been placed in the Upper Albian Stage of the Lower Cretaceous [19].

Of the two lamniform species found in both formations, *Leptostyrax macrorhiza* tend to be larger. In fact, teeth of this species represent some of the largest known shark fossils from the Albian of Texas, making it the most appealing suspect for the identification of OMNH 68860. However, these teeth are still generally smaller than those of the Late Cretaceous *Cretoxyrhina mantelli*, a species with known vertebral proportions similar to those of OMNH 68860 [15, 20]. However, biostratigraphic evidence suggests that the Texas vertebrae are not from *C*. *mantelli*, as teeth from this species do not appear in Texas until the Cenomanian [15]. Further comparisons of OMNH 68860 to those vertebrae of *C*. *mantelli* from the Niobrara Formation demonstrate stark differences.

Unlike most sharks, *C*. *mantelli* has a surprisingly complete fossil record, with multiple specimens preserving both teeth and postcranial material. Vertebrae from this species tend to be large, exhibiting typical lamnoid-type centra, with a pair of cartilage foramina for both the neural and haemal arches (personal observation). Numerous radiating lamellae are also present that progressively decrease in number caudally down the vertebral column. These vertebrae differ from OMNH 68860 in size, shape, and number of the cartilage foramina. Specifically these foramina in OMNH 68860 do not contact the corpus calcareum, are squarer, and are more numerous than in any published specimens of *C*. *mantelli* [11, 20].

Another Late Cretaceous lamniform shark with known postcranial material is *Cardabiodon ricki*. *C*. *ricki* was first described based on teeth and vertebrae from the Cenomanian of Australia [21]. Subsequently, teeth were also described from the Cenomanian of Kansas [22] and the Turonian of central Montana [23]. Superficially, vertebrae from *C*. *ricki* are more comparable to the Texas specimens than any of *C*. *mantelli*; for example, the vertebrae are more elongate, have a thick corpus calcareum, and small cartilage foramina. However, OMNH 68860 can be differentiated in that they have thinner and less densely spaced radial lamellae, square cartilage foramina, and concentric lamellae. These features, plus the absence of *Cardabiodon* teeth from the Albian of North America, make it unlikely that the Texas specimen belongs to this genus.

Aside from *Leptostyrax macrorhiza*, the only other lamniform shark known from both the Kiowa Shale and the Duck Creek Formation is *Cretalamna appendiculata*. The genus has a relatively long temporal range throughout the Cretaceous, with a worldwide distribution [24, 25]. In Texas, there is an apparent size shift in teeth of *C*. *appendiculata* through the Early to Late Cretaceous, where Albian teeth tend to be relatively small, but gradually increase until reaching their largest sizes (up to 30 mm) in the Maastrichtian. These larger Late Cretaceous specimens are considerably abundant in marine fossil sites [15].

**Fig 5:**
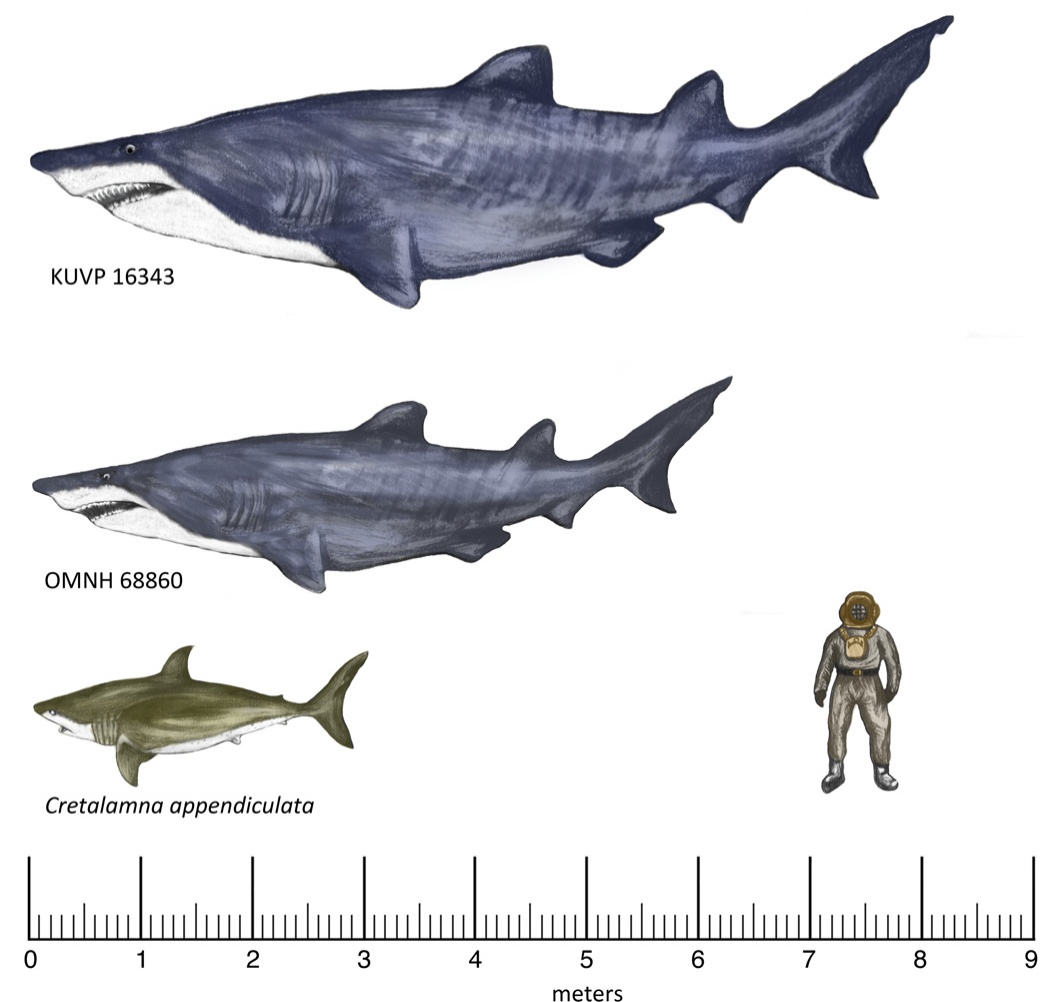
Reconstruction of the large lamniform sharks from the Duck Creek Formation and Kiowa Shale. KUVP 16343 and OMNH 68860 are both reconstructed as *Leptostyrax macrorhiza* and modeled after an odontaspidid. This reconstruction was based on dental similarities shared between Eoptolamnidae and Odontaspididae [14]. Both specimens represent the smallest calculated estimate based on the formula of Shimada [2]. *Cretalamna appendiculata* is reconstructed as a classic lamnid shark based on shared dental patterns between this genus and members of the family Lamnidae [26].

Shimada [26] described a relatively complete specimen (Los Angeles County Museum of Natural History [LACM] 128126) of *Cretalamna* from the Niobrara Formation (Upper Cretaceous). This specimen preserves the 35 anteriormost vertebral centra, with the largest measuring 48 mm in maximum diameter. These vertebrae are of typical lamnoid-form, with lamellae radiating around an amphicoelous centrum. The dorsal foramina are subovate and large, extending to meet the corpus calcareum on both sides. The ventral foramina also abut the corpus calcareum and possess smoothly reinforced edges, giving them a raised appearance [25]. This specimen differs substantially from OMNH 68860. For example, OMNH 68860 contains basidorsal and basiventral foramina that tend to be smaller and do not contact the corpus calcareum. Further, the raised edges of the ventral foramina are absent in this specimen. Lastly, most *C*. *appendiculata* tooth fossils from Texas are relatively small (up to 30 mm in height). Using LACM 128126 as an analog (maximum tooth length is 19.9 mm and vertebral width is 48 mm), a *Cretalamna* with the skeletal proportions of OMNH 68860 should have teeth between 45–50 mm in length. The only known species with teeth of this size from both the Kiowa Shale and the Duck Creek Formation is *Leptostyrax macrorhiza* (Fig 5).

Alternatively, a third lamniform species is recognized in the Albian of Texas and Kansas. *Carcharias amonensis*, an odontaspidid, has been reported from the upper Albian Paw Paw Formation and the lower Kiowa Shale [15, 27]. Although this species is not known from associated vertebral material, known teeth are relatively small (11 mm in maximum height [15]), making this species an unlikely candidate for the vertebrae. Given the absolute size and distinct morphology of OMNH 68860, it is highly unlikely that these vertebrae belong to any commonly known shark from the Duck Creek Formation, except possibly *Leptostyrax macrorhiza*.

Associated skeletal material is unknown for *L*. *macrorhiza*, but teeth are relatively abundant. In a recent reevaluation of the genus, Kriwet et al. [14] moved *L*. *macrorhiza* into a new family, Eoptolamnidae, along with the genera *Protolamna* and *Eoptolamna*. This family is characterized by a weak monognathic heterodonty, a single pair of symphyseal teeth, and high and robust anterior to lateral teeth that possess a well-developed nutritive groove. Interestingly, this group shares many similarities with multiple shark families, including Cretoxyrhinidae, Miitsukurinidae, and Odontaspididae. Unfortunately this diagnosis does not include vertebral characters, but leaves the possibility open that the unique anatomy of the Texas specimen is representative of the group. Further, given the unknown phylogenetic placement of these vertebrae, the size calculations produced here should be viewed as a coarse approximation to the actual size of the animal because lamniform families each possess different skeletal proportions. For example, a modern odontaspidid with a total length of 2.9 m has a largest vertebra equaling 36.6 mm [28]. Using this proportion, OMNH 68860 would have a total length of 8.9 m, or approximately 29% above the smallest calculated total length using Shimada’s [2] equation based on a cretoxyrhinid. Because of the uncertainties regarding the animal’s proportions in life and whether the known material actually represents the largest vertebra in the body, we must assume that the smallest estimates (6.3 m) represent a minimum size for this individual, with the likely possibility that these sharks could far exceed those estimates.

### Ecological Implications

Whether or not OMNH 68860 belongs to *Leptostyrax* remains unclear; however, the unique vertebral morphology and gigantic size indicate the presence of a very large shark during the mid-Cretaceous of North America. The mid-Cretaceous is increasingly being recognized as an important time in shark evolutionary history, as the fossil record improves and increasingly reveals previously unknown diversity. Although Lamniformes likely evolved in the Jurassic, it is not until the Aptian when multiple genera appear together in a single assemblage. Similarly, size increased for the entire order, with multiple families containing relatively large species by the Late Cretaceous [29]. This increase in size and diversity was likely influenced by the warming trend beginning in the mid-Cretaceous; where midocean temperatures at 30–35°N paleolatitude rise from 13–14°C in the early Albian to 28–29°C in the Cenomanian [30]. However, more research is needed to determine the cause of gigantism in lamniform sharks.

This discovery has further implications on the ecology of Mesozoic oceans. In modern oceans, many large lamniform sharks are apex pelagic predators of marine and nearshore ecosystems. Chondrichthyans the size of OMNH 68860 would be among the largest predatory animals of the Albian oceans, dwarfed only by some of the contemporaneous pliosaurs [31, 32]. Fossil tooth marks on dinosaurs [33], mosasaurs [34], plesiosaurs [35], teleost fishes [36], and turtles [37], indicate that large lamniform sharks of the Late Cretaceous occupied the ecological position of generalist predator and scavenger, much as they do today. The discovery of OMNH 68860 highlights an important ecological transition during the Albian, where lamniform sharks begin to take on the massive sizes and trophic abilities seen most predominately in later occurring species. Further, both OMNH 68860 and KUVP 16343 represent Albian species with minimum total lengths between 6.3–8.3 m. This suggests that the late Early Cretaceous was home to some of the largest Mesozoic lamniform sharks of North America.

## Conclusion

The principal conclusions of this paper are:

1. Large shark vertebrae were recovered from the Lower Cretaceous Duck Creek Formation of Texas. These vertebrae represent a single animal of approximately 6.3 m in minimum total length, making this individual one of the largest documented sharks from the Early Cretaceous of N. America.
2. This specimen has unique morphology undocumented in any other Cretaceous shark from North America, but shares large size with a contemporaneous vertebra from the Kiowa Shale of Kansas.
3. We hypothesize that these vertebrae belong to *Leptostyrax macrorhiza* based on their size and co-occurrence in both the Duck Creek Formation and Kiowa Shale. However, without associated teeth, this identification cannot be confirmed.
4. The Albian oceans contained some of the largest lamniform sharks of the Mesozoic, which hypothetically represented an ecological precursor to the large sharks of the Late Cretaceous and Cenozoic.
